# Editorial: Neuropharmacological, Neurobiological and Behavioral Mechanisms of Learning and Memory

**DOI:** 10.3389/fphar.2019.00218

**Published:** 2019-03-15

**Authors:** Alfredo Meneses, Antonella Gasbarri, Assunta Pompili

**Affiliations:** ^1^Centro de Investigación y de Estudios Avanzados (CINVESTAV), México City, Mexico; ^2^Department of Biotechnological and Applied Clinical Sciences, University of L'Aquila, L'Aquila, Italy

**Keywords:** drugs, behavior, memory tasks, clinical, humans, animals

In our initial call, we mentioned that memory is a basic function of the brain, and fundamental in our life. It might be defined according to its content, time, and neurobiological basis: in the former case, as declarative/explicit or non-declarative/implicit memory; regarding time, as short-term (STM) or working, and long-term memory (LTM); and the latter depends on protein and mRNA synthesis. We know now that based on its molecular changes memory, covers several phases and times (e.g., Izquierdo et al., 2006; Ben-Yakov et al., 2015; Asok et al., 2018). According with Asok et al. (2018) there has been important advances in identifying the electrophysiological, genetic, proteomic, and epigenetic underpinnings of long-term memory (LTM).

As the present Research Topic shows, the investigation of memory mechanisms and related brain areas represent one of the most important topics in neuroscience. Memory is a field of scientific investigation in constant expansion. It is unsurprising that thousands of papers already have been published dealing with this subject and we frequently find them in diverse journals, making difficult the identification of clear insights. An effective way to provide appropriate empiric and conceptual frames might be to make available compilations. With this aim, we are organizing the present Research Topic. Certainly, the exact mechanisms of memory remain promising subjects. Readers from preclinical to clinical backgrounds will find interesting neuropharmacological, neurobiological, and/or behavioral insights of the mechanisms of learning and memory, and more importantly contributions combining these approaches. Although we miss theoretical and historical analyses, the richness and variability of tools and approaches used in the papers might reveal key insights and will be a decisive step forward in this topic.

Moreover, attempting going beyond the “memory disorders” notion, the classic Alzheimer's disease and the present dominant behavioral memory tasks (see e.g., Arakawa and Iguchi, 2018) as was the Morris water maze, and considering that cognitive dysfunction occurs in diverse psychiatric disorders (e.g., Millan et al., 2012). For instance, what treatments might be useful for memory alterations component present in posttraumatic stress disorder (PTSD), Attention deficit/hyperactivity disorder (ADHD) and drug addiction? As it becomes clear that Neuropharmacological, Neurobiological and Behavioral Mechanisms are involved, then behavioral, enrichment environmental, and pharmacological manipulations will be necessary?

Although we are far away to our initial aim, however in this Ebook readers will find 36 papers covering original research papers and a few reviews using diverse behavioral memory tasks and approaches. We appreciate very much all authors and reviewers who were generous with their time. A few manuscripts were rejected, sometimes based on fair professionals comments. Almost all the times the editorial office worked hard to get a fair and balance decisions.

## Author Contributions

AM and AG organized and worked as referee. AP support as referee in several papers.

### Conflict of Interest Statement

The authors declare that the research was conducted in the absence of any commercial or financial relationships that could be construed as a potential conflict of interest.
